# Randomised Phase 1b/2 trial of tepotinib vs sorafenib in Asian patients with advanced hepatocellular carcinoma with MET overexpression

**DOI:** 10.1038/s41416-021-01380-3

**Published:** 2021-05-10

**Authors:** Baek-Yeol Ryoo, Ann-Li Cheng, Zhenggang Ren, Tae-You Kim, Hongming Pan, Kun-Ming Rau, Hye Jin Choi, Joong-Won Park, Jee Hyun Kim, Chia Jui Yen, Ho Yeong Lim, Dongli Zhou, Josef Straub, Juergen Scheele, Karin Berghoff, Shukui Qin

**Affiliations:** 1 Department of Oncology, Asan Medical Center, University of Ulsan College of Medicine, Seoul, Republic of Korea; 2 Department of Oncology, National Taiwan University Hospital, Taipei, Taiwan; 3 Liver Cancer Institute, Department of Hepatic Oncology, Zhongshan Hospital, Fudan University, Shanghai, China; 4 Department of Internal Medicine, Seoul National University Hospital, Seoul, Republic of Korea; 5 School of Medicine, Sir Run Run Shaw Hospital, Zhejiang University, Hangzhou, China; 6 Department of Internal Medicine, Kaohsiung Chang Gung Memorial Hospital and E-Da Cancer Hospital, Kaohsiung, Taiwan; 7 Department of Internal Medicine, Yonsei University College of Medicine, Seoul, Republic of Korea; 8 Center for Liver and Pancreatobiliary Cancer, National Cancer Center, Goyang-si, Republic of Korea; 9 Department of Internal Medicine, Seoul National University Bundang Hospital, Seoul National University College of Medicine, Seongnam-si, Republic of Korea; 10 Department of Internal Medicine, National Cheng Kung University Hospital, Tainan City, Taiwan; 11 Department of Medicine, Samsung Medical Center, Sungkyunkwan University, Seoul, Republic of Korea; 12 EMD Serono Research & Development Institute, Inc. (A Business of Merck KGaA, Darmstadt, Germany), Billerica, MA USA; 13 Clinical Biomarker & Companion Diagnostics, Merck KGaA, Darmstadt, Germany; 14 Clinical Oncology, Global Research and Development, Merck KGaA, Darmstadt, Germany; 15 Global Patient Safety Innovation, Merck KGaA, Darmstadt, Germany; 16 Medical Oncology Department, PLA Cancer Center, Nanjing Bayi Hospital, Nanjing, China

**Keywords:** Hepatocellular carcinoma, Molecularly targeted therapy

## Abstract

**Background:**

This open-label, Phase 1b/2 study evaluated the highly selective MET inhibitor tepotinib in systemic anticancer treatment (SACT)-naive Asian patients with advanced hepatocellular carcinoma (aHCC) with MET overexpression.

**Methods:**

In Phase 2b, tepotinib was orally administered once daily (300, 500 or 1,000 mg) to Asian adults with aHCC. The primary endpoints were dose-limiting toxicities (DLTs) and adverse events (AEs). Phase 2 randomised SACT-naive Asian adults with aHCC with MET overexpression to tepotinib (recommended Phase 2 dose [RP2D]) or sorafenib 400 mg twice daily. The primary endpoint was independently assessed time to progression (TTP).

**Results:**

In Phase 1b (*n* = 27), no DLTs occurred; the RP2D was 500 mg. In Phase 2 (*n* = 90, 45 patients per arm), the primary endpoint was met: independently assessed TTP was significantly longer with tepotinib versus sorafenib (median 2.9 versus 1.4 months, HR = 0.42, 90% confidence interval: 0.26–0.70, *P* = 0.0043). Progression-free survival and objective response also favoured tepotinib. Treatment-related Grade ≥3 AE rates were 28.9% with tepotinib and 45.5% with sorafenib.

**Conclusions:**

Tepotinib improved TTP versus sorafenib and was generally well tolerated in SACT-naive Asian patients with aHCC with MET overexpression.

**Trial registration:**

ClinicalTrials.gov NCT01988493.

## Background

Hepatocellular carcinoma (HCC) is the most common type of primary liver cancer in adults.^1^ Its incidence is rising alongside increasing rates of chronic liver disease. For many years, the only approved targeted systemic therapy for advanced HCC was the non-selective multikinase inhibitor sorafenib.^2,3^ This agent provides only a modest improvement in overall survival (OS) and may not be as well tolerated by Asian patients compared with those of other ethnicities.^4,5^ Newer first-line treatment options include the multikinase inhibitor lenvatinib and the immunotherapy atezolizumab (in combination bevacizumab), which have been approved following positive data from randomised Phase 3 trials versus sorafenib.^3,6–8^

MET is the tyrosine kinase receptor for hepatocyte growth factor (HGF).^9^ Approximately 50% of patients with HCC may harbour *MET* alterations,^9^ and 28% of patients with advanced HCC show evidence of MET overexpression.^10^ These patients may derive therapeutic benefit from selective MET inhibition.^9,11^ In vitro, MET inhibitors can reduce the growth of MET-positive HCC cell line-derived xenograft models.^11^

Tepotinib is an orally available, potent and highly selective MET inhibitor that has shown pronounced antitumour activity in MET-dependent preclinical mouse models in vivo.^12,13^ In a first-in-human study in US/European patients with various solid cancers (including HCC), tepotinib was generally well tolerated and demonstrated activity in tumours harbouring *MET* alterations.^13^ Tepotinib also showed durable clinical activity and was generally well tolerated in a Phase 2 study in patients with non-small cell lung cancer with *MET* exon 14 skipping, in which consistent activity was observed in the subgroup of Asian patients.^14,15^ Furthermore, in combination with gefitinib, tepotinib has demonstrated improved efficacy compared with chemotherapy in patients with advanced epidermal growth factor receptor-mutant non-small cell lung cancer and MET-driven resistance to epidermal growth factor receptor (EGFR) inhibitors^16^ and a Phase 2 study of tepotinib plus osimertinib in patients with acquired resistance to first-line osimertinib due to *MET* amplification is ongoing (INSIGHT 2, NCT03940703). A Phase 2 study is also underway investigating tepotinib in combination with cetuximab in patients with *RAS/BRAF* wild-type left-sided metastatic colorectal cancer and acquired resistance to anti-EGFR antibody-targeted therapy due to *MET* amplification (NCT04515394).

Following preclinical data demonstrating activity of tepotinib against primary liver cancer explants with MET overexpression,^12^ two Phase 1b/2 studies were designed to investigate tepotinib in patients with HCC with MET overexpression. In the first of these (NCT02115373), tepotinib demonstrated clinical activity and was generally well tolerated in US/European patients in whom prior sorafenib treatment had failed.^17^ Here, we present the second trial in HCC, which was conducted in Asian patients (NCT01988493). Since patients with hepatic impairment were excluded from the first-in-human trial,^13^ the trial included a Phase 1b part to establish the recommended Phase 2 dose (RP2D) of tepotinib in patients with HCC and Child–Pugh Class A liver function. The Phase 2 part of the trial then evaluated the activity and safety of the RP2D of tepotinib versus sorafenib in systemic anticancer treatment (SACT)-naive Asian patients with MET overexpression.

## Methods

### Study design and objectives

This was an open-label, multicentre, integrated, Phase 1b/2 trial conducted in Asian patients with advanced HCC (NCT01988493). The Phase 1b part of the trial was an open-label, single-arm, dose-escalation study with a classic ‘3 + 3’ design and a primary objective to establish the RP2D of tepotinib. The Phase 2 part was a multicentre, randomised, open-label, active-controlled study to evaluate the activity and safety of tepotinib as monotherapy versus sorafenib (Supplementary Fig. 1). Phase 1b was conducted at eight sites and Phase 2 at 43 sites in mainland China, South Korea and Taiwan.

All patients provided written informed consent for participation in the study. The study was done in accordance with the Declaration of Helsinki, International Council for Harmonization guideline for Good Clinical Practice, local laws and applicable regulatory requirements. The study was approved by the institutional review board or independent ethics committee of each centre.

### Patients

Patients were enrolled by the investigators. In both study phases, eligible patients were Asian and aged ≥18 years with histologically or cytologically confirmed HCC of Barcelona Clinic liver cancer (BCLC) stage B or C with Child–Pugh Class A liver function, and Eastern Cooperative Oncology Group performance status 0 or 1. Exclusion criteria included prior treatment with any agent targeting the HGF/MET pathway, prior local–regional therapy within 4 weeks before Day 1 of study treatment and presence of symptomatic or untreated brain metastases.

Based on a local regulatory requirement, patients enrolled in South Korea in Phase 1b were required to have experienced disease progression or intolerance to prior standard treatment for advanced HCC. In Phase 2, prior SACT for advanced HCC was not allowed. Eligibility for Phase 2 also required MET overexpression, as determined during molecular pre-screening or screening by immunohistochemistry (IHC) analysis of tumour biopsy samples using the pharmDx anti-total MET (D1C2) rabbit monoclonal antibody (Dako, Agilent Technologies, Inc., Santa Clara, CA, US) (Supplementary Table 1). MET overexpression was defined as moderate (2+) or strong (3+) staining for MET in ≥50% of tumour cells. Due to a quality issue in central MET IHC assessments detected during routine monitoring, central re-scoring of all MET IHC analyses was conducted after enrolment as a quality control measure. MET expression status assessment was not required for Phase 1b, but was determined retrospectively. In Phase 2, *MET* amplification was assessed retrospectively by fluorescence in situ hybridisation using the Dako *MET* IQFISH probe (Agilent Technologies, Inc.) and defined by mean *MET* gene copy number ≥5.^18^ Full inclusion and exclusion criteria are shown in Supplementary Table 2.

### Treatment administration

In Phase 1b, patients were treated orally, once daily (QD), in 21-day cycles with tepotinib hydrochloride hydrate 300 mg, 500 mg or 1,000 mg (containing 270, 450 and 900 mg, respectively, of the active moiety in free base form). The target RP2D was defined as 500 mg, based on the results of the first-in-human trial.^13^ Patients enrolled in South Korea and Taiwan received tepotinib according to a classic ‘3 + 3’ dose-escalation design at 300 mg or 500 mg. Once three patients had completed one cycle at 500 mg, nine further patients were to be enrolled at this dose level to confirm the RP2D. Patients were replaced if they discontinued during Cycle 1 for reasons other than a dose-limiting toxicity (DLT), or if they did not receive ≥80% of the planned dose during Cycle 1 for reasons other than DLTs or adverse events (AEs) related to tepotinib. The Safety Monitoring Committee (SMC) could also recommend enrolment of additional patients at or above the RP2D. Although no DLTs were observed, the SMC recommended the enrolment of a further three patients at 300 mg to provide additional data at this dose level. After the RP2D had been defined at 500 mg and in parallel with the Phase 2 part of the study, the SMC also recommended enrolment of six patients in South Korea to receive tepotinib at 1,000 mg, to enable characterisation of safety and pharmacokinetics of this dose. Separate from the ‘3 + 3’ dose-escalation cohorts, up to three patients were to be enrolled in mainland China to receive tepotinib 300 mg.

In Phase 2, patients were treated with tepotinib orally QD at the RP2D, or with sorafenib 400 mg orally twice daily. Patients in both phases continued to receive allocated therapy until disease progression, intolerable toxicity or withdrawal of consent.

### Randomisation and masking

In Phase 2, patients were randomly allocated to receive tepotinib or sorafenib in a 1:1 ratio. The randomisation sequence was computer-generated by the sponsor and implemented via an interactive voice-response system (which also concealed the sequence). A stratified, permuted block randomisation procedure (block size of 6, with sub-block sizes of 4 and 2) was used with BCLC stage B versus C as the strata criteria. Neither clinicians nor patients were blinded to treatment selection.

### Study endpoints and assessments

In Phase 1b, the primary endpoint was the incidence of investigator-assessed DLTs occurring in Cycle 1, as well as the incidence of other AEs (graded according to the National Cancer Institute Common Terminology and Criteria for Adverse Events, v4.0). DLTs are defined in Supplementary Table 3. Secondary endpoints in Phase 1b included efficacy measures and pharmacokinetic parameters, such as C_max_ and area under the concentration–time curve over the dosing interval at steady state (AUC_τ,ss_), which were evaluated at Cycle 1, Day 15.

In Phase 2, the primary endpoint was independent review committee (IRC)-assessed time to progression (TTP) per Response Evaluation Criteria in Solid Tumors (RECIST) v1.1. Secondary endpoints included investigator-assessed TTP, IRC- and investigator-assessed progression-free survival (PFS), objective response rate (ORR), disease control rate, OS and safety. In both phases, tumour assessments were performed according to RECIST v1.1 based on scans taken at the end of every second cycle until Cycle 13, and every four cycles thereafter.

### Statistical analysis

Phase 1b data were analysed in a descriptive manner; DLTs were assessed in all patients who experienced a DLT during Cycle 1 or completed at least 80% of planned treatment during the 21 days after the first dose of tepotinib.

In Phase 2, analyses were conducted according to randomised treatment assignment. TTP was analysed in a modified intention-to-treat (mITT) population, which included all patients randomised to study treatment for whom MET overexpression was confirmed during planned re-scoring of IHC analyses (i.e. patients with MET status 1+ or ‘not assessable’ on re-scoring were excluded from the mITT population). In total, 100 TTP events were required to ensure 80% power (with a two-sided significance level of 10%) for rejecting the null hypothesis of equal treatment effect between treatment arms, assuming a true hazard ratio (HR) of 0.6. With an assumed accrual period of 12 months, follow-up period of 6 months and overall drop-out rate of 17.4%, 140 patients with HCC with MET overexpression were planned to be enrolled. The HR (including 90% confidence interval [CI], calculated by Cox’s proportional hazards model, stratified by BCLC stage) and Kaplan–Meier survival estimates were used to compare TTP, PFS and OS between tepotinib and sorafenib. For the primary and secondary time-to-event endpoints, treatment groups were compared in the mITT population, applying a two-sided log-rank test, stratified by BCLC stage (α = 10%). Safety analyses in Phase 2 were conducted in all patients who received at least one dose of study medication. The data cut-off was May 31, 2017, for Phase 1b and March 12, 2018, for Phase 2.

## Results

### Patients and treatment

Patients were enrolled between February 2014 and August 2017. Of 41 patients screened in Phase 1b (Supplementary Fig. 2a), 27 patients were recruited. All patients had discontinued treatment at data cut-off. Baseline characteristics are shown in Supplementary Table 4. The 300 mg cohort comprised six patients who received tepotinib in the dose-escalation phase and one patient enrolled in mainland China. The 500 mg cohort included three patients from the dose-escalation phase and nine from the dose-confirmation phase. A further two patients were enrolled at 500 mg to replace patients not evaluable for DLTs (one due to Grade 2 bacteraemia, unrelated to tepotinib, and one due to disease progression). Six patients were enrolled at the 1,000 mg dose level.

Enrolment into Phase 2 was stopped early due to slow accrual. A total of 619 patients entered pre-screening and 151 patients were screened for eligibility (Supplementary Fig. 2b). Of 592 patients assessed by IHC at pre-screening or screening, 161 (27.2%) had MET overexpression in initial scoring. Ninety patients were randomised to treatment (tepotinib, *n* = 45; sorafenib, *n* = 45), which was lower than the enrolment target of 140 patients. One patient randomised to sorafenib did not receive any study treatment. After the exclusion of 15 patients who did not have MET overexpression on planned re-scoring of IHC analyses, the mITT population included 38 and 37 patients in the tepotinib and sorafenib arms, respectively. Baseline characteristics were generally similar between the tepotinib and sorafenib treatment arms in Phase 2, although more patients in the sorafenib arm had MET IHC 3+ status (35.1%) compared with the tepotinib arm (5.3%) (Table 1). Median duration of tepotinib therapy was 12.7 weeks (interquartile range, 6.1–26.3), and median duration of sorafenib therapy was 11.9 weeks (interquartile range, 6.1–16.6). After discontinuation of study treatment, subsequent anticancer therapy was administered in 11 patients (28.9%) in the tepotinib arm (including sorafenib in 10 patients, 26.3%) and 17 patients (45.9%) in the sorafenib arm.

**Table 1:** Baseline characteristics (Phase 2 study; mITT population^a^).

	Tepotinib	Sorafenib	Total
	*n* = 38	*n* = 37	*n* = 75
Median (range) age, years	59 (38–78)	54 (31–78)	57 (31–78)
Aged <65 years, *n* (%)	31 (81.6)	32 (86.5)	63 (84.0)
Male, *n* (%)	37 (97.4)	34 (91.9)	71 (94.7)
Region, *n* (%)			
Mainland China	14 (36.8)	12 (32.4)	26 (34.7)
Republic of Korea	18 (47.4)	19 (51.4)	37 (49.3)
Taiwan	6 (15.8)	6 (16.2)	12 (16.0)
Prior local–regional anticancer therapy, *n* (%)			
Yes	20 (52.6)	20 (54.1)	40 (53.3)
No	18 (47.4)	17 (45.9)	35 (46.7)
HBV test, *n* (%)			
Positive	24 (63.2)	30 (81.1)	54 (72.0)
Negative	10 (26.3)	6 (16.2)	16 (21.3)
Missing	4 (10.5)	1 (2.7)	5 (6.7)
HCV test, *n* (%)			
Positive	2 (5.3)	5 (13.5)	7 (9.3)
Negative	22 (57.9)	21 (56.8)	43 (57.3)
Missing^b^	14 (36.8)	11 (29.7)	25 (33.3)
HBV/HCV at baseline, *n* (%)			
Either positive	24 (63.2)	30 (81.1)	54 (72.0)
Both negative or one negative/one missing	10 (26.3)	6 (16.2)	16 (21.3)
Both missing	4 (10.5)	1 (2.7)	5 (6.7)
Alcohol use, *n* (%)			
Never	13 (34.2)	14 (37.8)	27 (36.0)
Regular	5 (13.2)	4 (10.8)	9 (12.0)
Occasional	0 (0.0)	1 (2.7)	1 (1.3)
Former	20 (52.6)	18 (48.6)	38 (50.7)
AFP, *n* (%)			
≥200 IU/mL	22 (57.9)	24 (64.9)	46 (61.3)
<200 IU/mL	16 (42.1)	13 (35.1)	29 (38.7)
Vascular invasion, *n* (%)			
Yes	12 (31.6)	15 (40.5)	27 (36.0)
No	16 (42.1)	6 (16.2)	22 (29.3)
Missing	10 (26.3)	16 (43.2)	26 (34.7)
BCLC stage, *n* (%)			
B	2 (5.3)	2 (5.4)	4 (5.3)
C	36 (94.7)	35 (94.6)	71 (94.7)
MET IHC, *n* (%)			
IHC 2+	36 (94.7)	24 (64.9)	60 (80.0)
IHC 3+	2 (5.3)	13 (35.1)	15 (20.0)
*MET* amplification,^c^ *n* (%)			
Present	4 (10.5)	5 (13.5)	9 (12.0)
Absent	32 (84.2)	32 (86.5)	64 (85.3)
Missing	2 (5.3)	0 (0.0)	2 (2.7)

*AFP* alpha-fetoprotein, *BCLC* Barcelona Clinic liver cancer, *GCN* gene copy number, *HBV* hepatitis B virus, *HCV* hepatitis C virus, *IHC* immunohistochemistry, *IU* international units, *mITT* modified intention-to-treat.

^a^mITT excludes patients that were MET IHC 1+ or not assessable based on re-scoring.

^b^HCV testing was a late addition to the study protocol; therefore, HCV is missing for 25/75 patients.

^c^*MET* amplification defined as mean GCN ≥ 5.

### Safety

No DLTs were established with any dose of tepotinib in Phase 1b, and, therefore, the RP2D of tepotinib was established as 500 mg QD. All patients who received tepotinib in both Phase 1b (*n* = 27) and Phase 2 (*n* = 45) experienced AEs of any cause. In the sorafenib arm of Phase 2 (*n* = 44), 43 patients (97.7%) experienced AEs of any cause. In Phase 2, the most common AEs of any cause with tepotinib were peripheral oedema (42.2%), diarrhoea (37.8%) and decreased appetite (35.6%), and with sorafenib were palmar–plantar erythrodysesthesia (61.4%), decreased appetite (40.9%), diarrhoea (38.6%) and aspartate transaminase increase (36.4%).

In Phase 1b, 55.6% of patients receiving tepotinib experienced Grade ≥3 AEs of any cause. In Phase 2, 60% of patients receiving tepotinib and 70.5% of patients receiving sorafenib experienced Grade ≥3 AEs of any cause. In Phase 2, permanent treatment discontinuation due to AEs of any cause occurred in seven patients (15.6%) receiving tepotinib: these AEs were ascites (*n* = 1), upper gastrointestinal haemorrhage (*n* = 1), fatigue (*n* = 2), hepatic failure (*n* = 1), QT interval prolongation (*n* = 1), and hepatic encephalopathy (*n* = 1). Six patients (13.6%) receiving sorafenib permanently discontinued treatment due to AEs of any cause. Six patients (13.3%) receiving tepotinib in Phase 2 died due to AEs of any cause. This was deemed to be treatment-related in one instance of upper gastrointestinal haemorrhage. Two patients receiving sorafenib died due to AEs while on treatment; neither were deemed treatment-related.

In Phase 2, AEs considered to be related to study treatment by the investigators were reported in 37 (82.2%) of patients receiving tepotinib and 43 (97.7%) of patients receiving sorafenib. Grade ≥3 treatment-related AEs occurred in 13 (28.9%) and 20 (45.5%) patients receiving tepotinib and sorafenib, respectively (Table 2). The most common treatment-related AEs in patients receiving tepotinib were diarrhoea (35.6%), peripheral oedema (24.4%) and fatigue (20.0%). For patients receiving sorafenib, the most common treatment-related AEs were palmar–plantar erythrodysesthesia (61.4%), diarrhoea (31.8%) and decreased appetite (27.3%). The incidence of treatment-related AEs was similar in Phase 1b, with any-grade events observed in 22 (81.5%) and Grade ≥3 events in nine (33.3%) patients receiving tepotinib (across all dose cohorts).

**Table 2:** Treatment-related adverse events reported in ≥10% of patients (Phase 2 study; safety analysis set).

Patients with treatment-related adverse events, *n* (%)	Tepotinib		Sorafenib	
	*n* = 45		*n* = 44^a^	
	Any grade	Grade ≥ 3^b^	Any grade	Grade ≥ 3^b^
Overall	37 (82.2)	13 (28.9)	43 (97.7)	20 (45.5)
Diarrhoea	16 (35.6)	2 (4.4)	14 (31.8)	3 (6.8)
Oedema peripheral	11 (24.4)	0 (0.0)	0 (0.0)	0 (0.0)
Fatigue	9 (20.0)	2 (4.4)	11 (25.0)	0 (0.0)
PPES	8 (17.8)	1 (2.2)	27 (61.4)	3 (6.8)
Decreased appetite	8 (17.8)	0 (0.0)	12 (27.3)	0 (0.0)
Blood creatinine increased	6 (13.3)	0 (0.0)	0 (0.0)	0 (0.0)
AST increased	5 (11.1)	2 (4.4)	10 (22.7)	3 (6.8)
Hypoalbuminaemia	5 (11.1)	0 (0.0)	2 (4.5)	0 (0.0)
ALT increased	4 (8.9)	0 (0.0)	7 (15.9)	0 (0.0)
Amylase increased	3 (6.7)	2 (4.4)	5 (11.4)	1 (2.3)
Blood bilirubin increased	2 (4.4)	1 (2.2)	8 (18.2)	2 (4.5)
Alopecia	1 (2.2)	0 (0.0)	10 (22.7)	0 (0.0)
Lipase increased	1 (2.2)	0 (0.0)	5 (11.4)	4 (9.1)
Hypertension	0 (0.0)	0 (0.0)	11 (25.0)	6 (13.6)
Dermatitis acneiform	0 (0.0)	0 (0.0)	5 (11.4)	0 (0.0)

*ALT* alanine aminotransferase, *AST* aspartate aminotransferase, *PPES* palmar–plantar erythrodysesthesia syndrome.

^a^One patient did not receive treatment.

^b^Grade ≥ 3 treatment-related adverse events (in ≥ 2 patients) also included ascites (4.4%) and hyperglycaemia (4.4%) for tepotinib, and increased gamma-glutamyl transferase (4.5%) for sorafenib.

### Efficacy

**Fig. 1:**
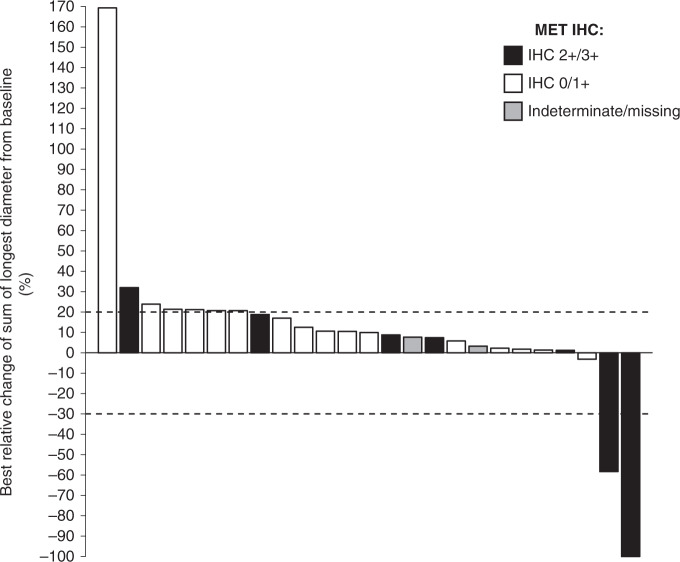
Relative change in sum of longest diameters of tumour lesions from baseline to post-baseline nadir for RECIST-evaluable patients (*n* = 25, Phase 1b study). *IHC* immunohistochemistry.

In Phase 1b, the best overall response was partial response (PR) in two patients: one in the 500 mg cohort (ORR, 7.1%) and one in the 1,000 mg cohort (ORR, 16.7%). Durations of PR were 19 months and 4.4 months, respectively. Tumour shrinkage was generally greater in patients with HCC with MET overexpression; both patients who achieved PR had HCC with MET overexpression (*n* = 1, IHC 2+, 1,000 mg cohort, and *n* = 1, IHC 3+, 500 mg cohort) (Fig. 1). The disease control rate was 50% in the 500 mg and 1,000 mg dose cohorts, and 14.3% in the 300 mg cohort. In the 300 mg, 500 mg and 1,000 mg cohorts, most patients had a best overall response of stable disease (14.3%, 42.9% and 33.3%, respectively) or progressive disease (85.7%, 35.7% and 50.0%, respectively). ORRs and disease control rates in Phase 1b suggested that efficacy increased with increasing dose.

**Fig. 2:**
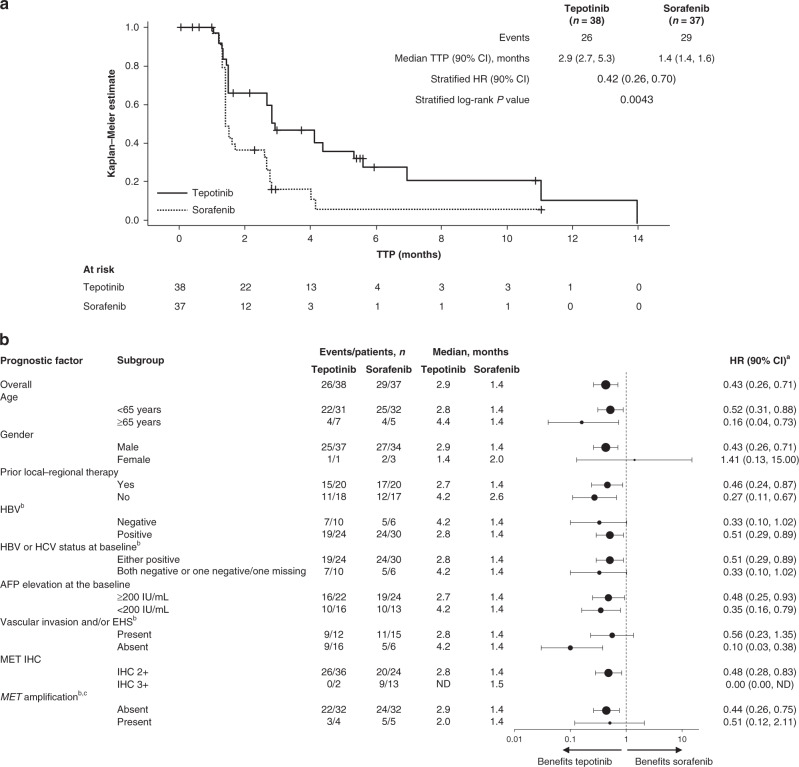
TTP assessed by IRC (Phase 2 study, mITT analysis set). **a** Kaplan–Meier curve. **b** Forest plot showing predefined subgroup analyses. ^a^Unstratified; ^b^Information not available for some patients in the tepotinib and/or sorafenib group; ^c^*MET* amplification defined as mean GCN ≥ 5. *AFP* alpha-fetoprotein, *CI* confidence interval, *EHS* extrahepatic spread, *GCN* gene copy number, *HBV* hepatitis B virus, *HCV* hepatitis C virus, *HR* hazard ratio, *IHC* immunohistochemistry, *IU* international units, *mITT* modified intent-to-treat, *ND* not determined, *TTP* time to progression.

The Phase 2 part of the study met its primary endpoint by demonstrating a significant improvement in IRC-assessed TTP in patients treated with tepotinib versus sorafenib (HR = 0.42, 90% CI: 0.26–0.70, *P* = 0.0043) (Fig. 2a). Median IRC-assessed TTP in the tepotinib arm was 2.9 months (90% CI: 2.7–5.3) versus 1.4 months (90% CI: 1.4–1.6) in the sorafenib arm. In pre-planned subgroup analyses, there was a benefit for tepotinib over sorafenib for every subgroup, except for the small number of female patients (Fig. 2b). Investigator-assessed TTP improved for tepotinib (median, 5.6 months; 90% CI: 3.0–7.6) versus sorafenib (median, 2.8 months; 90% CI: 1.5–2.8 months, HR = 0.45, 90% CI: 0.28–0.73, *P* = 0.0059).

**Fig. 3:**
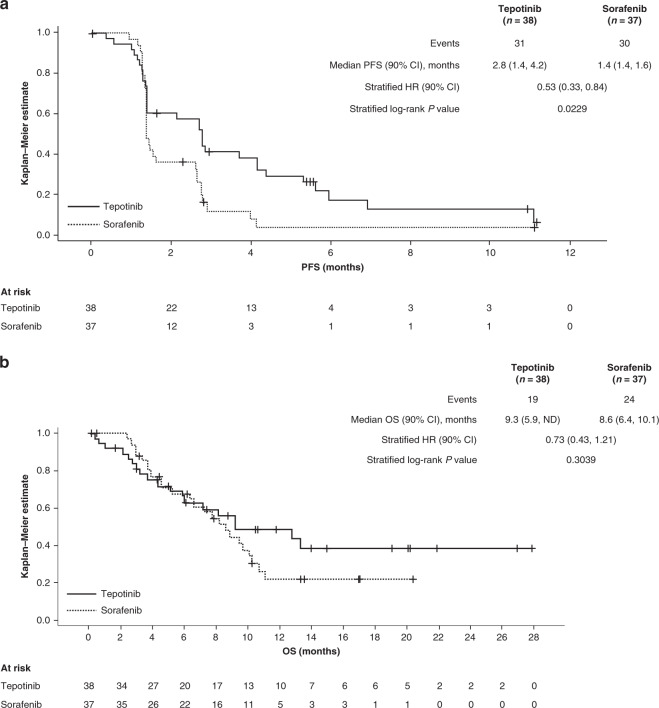
Kaplan–Meier curves for the Phase 2 study (mITT analysis set). **a** PFS assessed by IRC. **b** OS. *CI* confidence interval, *HR* hazard ratio, *IRC* independent review committee, *mITT* modified intention-to-treat, *ND* not determined, *OS* overall survival, *PFS* progression-free survival.

An improved PFS assessed by IRC was observed in patients treated with tepotinib versus sorafenib (HR = 0.53, 90% CI: 0.33–0.84, *P* = 0.0229) (Fig. 3a). Median PFS in the tepotinib arm was 2.8 months (90% CI: 1.4–4.2) versus 1.4 months (90% CI: 1.4–1.6) in the sorafenib arm. The results for investigator-assessed PFS were also improved with tepotinib compared with sorafenib (median PFS was 3.2 [90% CI: 2.7–5.6] versus 2.8 [90% CI: 1.5–2.8] months, respectively; HR = 0.59, 90% CI: 0.38–0.92, *P* = 0.0496). OS was similar between the study arms, with a median OS of 9.3 months and 8.6 months in the tepotinib and sorafenib arms, respectively (HR = 0.73, 90% CI: 0.43–1.21) (Fig. 3b).

The ORR by IRC was 10.5% in the tepotinib arm versus 0% in the sorafenib treatment arm (*P* = 0.0438) (Table 3). Four patients (10.5%) in the tepotinib arm had a best overall response of PR versus no patients in the sorafenib arm; no patients had a best overall response of complete response. As of Sept 2020, one patient with MET IHC 3+, *MET* amplification and a PR was still receiving treatment with tepotinib (treatment duration >45 months). Disease control was achieved by 19 patients (50%) and eight patients (21.6%) in the tepotinib and sorafenib arms, respectively.

**Table 3:** Best overall response as determined by IRC (Phase 2 study; mITT analysis set).

	Tepotinib	Sorafenib
	*n* = 38	*n* = 37
Best overall response		
Complete response	0 (0.0)	0 (0.0)
Partial response	4 (10.5)	0 (0.0)
Non-complete response/non-partial response^a^	2 (5.3)	0 (0.0)
Stable disease	15 (39.5)	8 (21.6)
Progressive disease	13 (34.2)	25 (67.6)
Not evaluable	4 (10.5)	4 (10.8)
Objective response rate, *n* (%)	4 (10.5)	0 (0.0)
90% CI,^b^ %	4.8, 21.5	0.0, 6.8
*P* value (CMH test)	0.0438	
Disease control rate, *n* (%)	19 (50.0)	8 (21.6)
90% CI,^b^ %	37.1, 62.9	12.6, 34.5

*CI* confidence interval, *CMH* Cochran–Mantel–Haenszel, *IRC* independent review committee, *mITT* modified intention-to-treat.

^a^Non-complete response/non-partial response was defined as persistence of one or more non-target lesion(s) and/or maintenance of tumour marker level, if measured, above the normal limits (possible only for patients without measureable disease at baseline).

^b^90% CI using the Newcombe–Wilson method.

### Pharmacokinetics

In Phase 1b, AUC_τ,ss_ of tepotinib was 11,800 ng*h/mL (35.7%) for the 300 mg dose group, 16,700 ng*h/mL (29.7%) for the 500 mg dose group and 28,600 ng*h/mL (38.8%) for the 1,000 mg dose group (figures are geometric mean and geometric coefficient of variation). Corresponding values for C_max_ of tepotinib were 585 ng/mL (30.8%), 815 ng/mL (31.6%) and 1,370 ng/mL (36.3%), respectively.

## Discussion

The Phase 1b portion of the present study confirmed the RP2D of tepotinib for the treatment of Asian patients with advanced HCC to be 500 mg daily. This same dose level has been established as the RP2D in other settings and patient populations, including US/European patients with solid tumours (the first-in-human study),^13^ Japanese patients with solid tumours^19^ and US/European patients with advanced HCC.^20^ Tepotinib also showed preliminary evidence of antitumour activity in the Phase 1b part of the study. In Phase 2, first-line tepotinib demonstrated clinical activity and was generally well tolerated in Asian patients with advanced HCC with MET overexpression (IHC 2+/3+). The primary endpoint was met, with a statistically significant improvement in TTP as assessed by IRC with tepotinib versus sorafenib. TTP benefit with tepotinib was seen across subgroups, although patient numbers were small in some subgroups. Greater antitumour activity of tepotinib versus sorafenib was also shown in terms of investigator-assessed TTP, as well as PFS and ORR.

Prior trials of inhibitors with activity against the MET receptor in advanced HCC have produced mixed findings. The non-selective MET inhibitor cabozantinib has been approved for use post-sorafenib, following the results from a placebo-controlled Phase 3 trial in MET-unselected HCC.^21^ In contrast, despite positive Phase 2 data,^22^ two Phase 3 trials of tivantinib in sorafenib pre-treated HCC with MET overexpression did not meet their primary endpoints.^23,24^ The selective MET inhibitor capmatinib has demonstrated an ORR of 10% in a small, single-arm Phase 2 first-line study of 30 patients with HCC with *MET* alterations (defined by MET H-score ≥ 50, *MET*:*CEP7* ratio ≥ 2.0 or *MET* gene copy number ≥ 5).^25^ While differences in trial design and settings prevent direct comparison, differences in the pharmacologic characteristics of the MET inhibitors, evaluated to date, could be relevant for clinical activity.^9^ For example, tepotinib, cabozantinib and capmatinib inhibit MET with in vitro IC_50_ values in the low nanomolar range, whereas tivantinib has a lower affinity for the receptor.^9^ Furthermore, unlike tepotinib and capmatinib, cabozantinib inhibits several other kinases and tivantinib has antimitotic effects, which may result from inhibition of glycogen synthase kinase-3α/β.^9,26,27^ Although inhibition of other pathways could contribute to anticancer effects, it may also constrain the maximum tolerated dose by increasing toxicity, and thereby limit clinical activity.^28^ Overall, the efficacy and safety of tepotinib shown in the present study lends further support to the strategy of selective MET inhibition in advanced HCC.

Subgroup analyses from the capmatinib study^25^ suggest more stringent definitions for *MET* alteration could predict greater benefit from selective MET inhibitors. A possible trend for greater 12-week PFS rates with tepotinib in patients with MET IHC 3+ status (versus 2+) or *MET* amplification (versus no *MET* amplification) was also observed in the Phase 1b/2 second-line trial.^17^ In the present study, the low number of patients with MET IHC 3+ staining in the tepotinib arm precludes assessment of the impact of MET IHC status (2+ versus 3+) on the activity of tepotinib versus sorafenib. Similarly, few patients with *MET* amplification were enrolled, although the subgroup analysis suggested that the TTP increase with tepotinib relative to sorafenib was similar in these patients compared with patients without *MET* amplification. The efficacy of tepotinib also appeared consistent, irrespective of alpha-fetoprotein elevation, which is a predictive marker for ramucirumab in the second-line setting.^29,30^

The median TTP of 1.4 months with sorafenib in the control arm of the present study is considerably lower than the 5.5 months in the Phase 3 SHARP sorafenib trial,^5^ and somewhat lower than the 2.8 months in the Phase 3 Asia-Pacific sorafenib trial.^4^ As in the Asia-Pacific trial, median TTP in the present study may have been impacted by the greater representation of patients with BCLC stage C (96%) relative to SHARP (82%).^2,4,5^ Shorter TTP with sorafenib could also reflect the high proportion of patients in the control arm of our study with MET IHC 3+ status (35.1%). Biomarker data from SHARP showing better TTP and OS with sorafenib in patients with lower (versus higher) baseline HGF^31^ suggest that over-activation of the MET signalling pathway may result in poor outcomes with sorafenib. MET IHC status in the present study was imbalanced between arms, and the low number of patients with 3+ status in the tepotinib arm could also have attenuated efficacy of this agent, given the evidence for association between more stringent definitions of *MET* alteration and better outcomes with selective MET inhibitors.^17,25^

Across both phases, tepotinib was generally well tolerated with no new safety signals. No DLTs were observed at any dose in Phase 1b. The incidence of treatment-related AEs was in line with previously published data,^13,15,17,19^ and no unexpected AEs were reported. Patients treated with tepotinib reported fewer overall and Grade ≥3 treatment-related AEs compared with sorafenib.

With the 500 mg dose in the Phase 1b study, tepotinib AUC_τ_ was 61% and C_max_ was 63% of that observed in the first-in-human trial.^13^ This is expected given findings from a population pharmacokinetic analysis showing a reduction in tepotinib exposure in patients with cirrhosis, as well as from a dedicated pharmacokinetic hepatic impairment trial.^32^ In the latter study, patients with moderate hepatic impairment (Child–Pugh B) had 12% lower AUC from time 0 to infinity and 29% lower C_max_ compared with control subjects without hepatic impairment. Lower exposure relative to the first-in-human trial was also observed in the Phase 1b/2 study in sorafenib pre-treated advanced HCC with MET overexpression.^33^

The selection of sorafenib, as the control treatment, for the present study reflects the standard of care for first-line treatment at the time of study conception. Since then, both the multikinase inhibitor lenvatinib and the combination of atezolizumab (anti-programmed death-ligand 1 [PD-L1]) plus bevacizumab (anti-vascular endothelial growth factor [VEGF]) have been approved in the first-line setting.^6,7^ After sorafenib failure, approved options include the anti-VEGF receptor-2 agent ramucirumab (for patients with elevated serum alpha-fetoprotein), the multikinase inhibitors regorafenib and cabozantinib and the immune checkpoint inhibitors pembrolizumab and nivolumab (± ipilimumab).^21,30,34–38^ While durable responses to nivolumab were observed in both sorafenib-naive and sorafenib pre-treated patients in the Phase 1/2 CheckMate 040 study,^37,39^ first-line nivolumab did not show an OS benefit compared with sorafenib in the Phase 3 CheckMate 459 trial.^40^ Other immunotherapy-containing strategies currently undergoing Phase 3 evaluation in previously untreated disease include durvalumab (anti-PD-L1), alone or in combination with a second immunotherapy (tremelimumab, anti-cytotoxic T-lymphocyte-associated protein-4; NCT03298451), lenvatinib plus pembrolizumab (LEAP-002; NCT03713593) and cabozantinib plus atezolizumab (COSMIC-312;^41^ NCT03755791). Given the favourable safety profile of tepotinib and the immunosuppressive function of MET signalling,^42^ use of tepotinib in combination with immunotherapies could be an interesting area for future study.

Strengths of the current study include the randomised design, which permitted evaluation of tepotinib efficacy relative to an established standard of care in this setting. Limitations include the non-blinded treatment assignment and the low proportions of patients with *MET* amplification or MET IHC 3+ status, which limited explorations of the impact of MET-based biomarkers on efficacy. The study was also underpowered following early termination of enrolment due to slow accrual, but nonetheless demonstrated significant improvements in activity endpoints with tepotinib versus sorafenib. As has been discussed for other studies in HCC with biomarker-driven patient selection,^23,24^ one challenge in this setting is the potential for patients with the most aggressive forms of disease to be excluded, due to rapid progression and/or clinical deterioration, while central biomarker assessments are ongoing, which could have contributed to ineligibility at screening. Finally, it is not known to what extent these results obtained in Asian patients, who were predominantly male with BCLC stage B, can be generalised to other populations.

In the Phase 1b part of this study, no DLTs were reported and the RP2D was established as 500 mg QD. Evidence of antitumour activity was seen at the 500 mg and 1,000 mg dose levels. In Phase 2, first-line tepotinib (500 mg QD) demonstrated clinical activity in Asian patients with advanced HCC with MET overexpression (IHC 2+/3+), with a significant improvement versus sorafenib in the primary endpoint of TTP, as well as PFS and ORR. Tepotinib was generally well tolerated and no new safety signals were observed.

## Supplementary information

Supplementary Information

## Data Availability

Data are held by the sponsor Merck KGaA (Darmstadt, Germany), to whom any request for additional data should be addressed.
