# Supplementary material for: Randomised Phase 1b/2 trial of tepotinib vs sorafenib in Asian patients with advanced hepatocellular carcinoma with MET overexpression
Source: Br J Cancer. 2021 May 10;125(2):200–8. doi: 10.1038/s41416-021-01380-3 (PMC8292411; doi:10.1038/s41416-021-01380-3)
Supplement: Supplementary file 1 — Supplementary Information [file 41416_2021_1380_MOESM1_ESM.docx]

### SUPPLEMENTARY INFORMATION

**Supplementary Table 1**. MET IHC antibodies

| **Phase** | **Antibody** | **Clone** | **Manufacturer** | **Quality control** |
| --- | --- | --- | --- | --- |
| Ib | CONFIRM anti-total MET rabbit monoclonal primary antibody | SP44 | Ventana Medical Systems, Inc., Oro Valley, AZ | Each MET IHC assay had positive and negative tissue controls, as well as a negative reagent control for each specimen that was included in each staining run. If there was failure of any one of the controls, patient results were considered invalid and the test was rerun. The pathologist reviewed all the control slides prior to scoring patient samples. |
| II | Dako pharmDx anti-total MET rabbit monoclonal antibody | D1C2 | Dako, Agilent Technologies, Inc., Santa Clara, CA |  |

*IHC* immunohistochemistry.

**Supplementary Table 2**. **a** Inclusion and **b** exclusion criteria

**a**

| **Inclusion criteria** | |
| --- | --- |
| - Histologically or cytologically confirmed HCC - Intermediate HCC of BCLC stage B, who were not eligible for surgical and/or local-regional therapies* or who had PD after surgical and/or local-regional therapies*, or advanced HCC of BCLC stage C - A tumor biopsy (excluding fine-needle aspiration and cytology samples) was required for determining MET status (a fresh pretreatment tumor biopsy was recommended but an archived tumor sample was acceptable) - Child–Pugh class A with no encephalopathy at screening - Asian male or female patients ≥18 years of age. - ECOG PS of 0 or 1 - Signed and dated informed consent indicating that the patient had been informed of all the pertinent aspects of the study before enrollment - Willingness and ability to comply with scheduled visits, treatment plans, laboratory tests, and other study procedures - Life expectancy of ≥3 months as judged by the investigator | |
| **Phase Ib only** | **Phase II only** |
| - PD or intolerance to prior standard treatment for advanced HCC (Korean patients only) | - MET overexpression, as determined by the central laboratory, defined according to MET protein staining by IHC (e.g. moderate [2+] or strong [3+] staining intensity in ≥50% of tumor cells) - Measurable disease in accordance with RECIST v1.1. The target lesion that had received previous local therapy was not to be considered as measurable unless clear progression had been documented since the therapy - Eligible for treatment with sorafenib, as assessed by investigators according to the Package Insert and clinical judgment |

**b**

| **Exclusion criteria** | |
| --- | --- |
| - Prior treatment with any agent targeting the HGF/MET pathway - Prior local-regional therapy within 4 weeks prior to day 1 of study treatment (e.g. major surgery, radiation therapy (with the exception of palliative bone-directed radiotherapy^†^ and radiotherapy administered to superficial lesions), hepatic arterial embolization, transcatheter arterial chemoembolization, chemoembolization, radiofrequency ablation, percutaneous ethanol injection, or cryoablation) - History of liver transplant. - Laboratory index at baseline:   - Hemoglobin ≤8.5 g/dL (without transfusion or growth factor support in the preceding 14 days)   - Neutrophils <1.5 × 10^9^/L   - Platelets <60 × 10^9^/L (without transfusion or growth factor support in the preceding 7 days)   - Total bilirubin >3 mg/dL   - Aspartate aminotransferase/alanine aminotransferase >5 × ULN   - Renal impairment as evidenced by serum creatinine ≥1.5 × ULN, or calculated CrCl <60 mL/min by Cockroft–Gault formula (24-hour CrCl could be requested by the investigator for confirmation, if calculated CrCl was <60 mL/min. In such cases, patients with 24-hour CrCl <60 mL/min were to be excluded)   $CrCl \left( {ml\cdot min}^{-1} \right)= \frac{140-age \left( years \right) \times weight (kg)}{72 \times serum creatinine (mg\cdot{dL}^{-1})} [\times0.85 for women]$   - - International normalized ratio >2.3 (in accordance with the guidance that was modified for Child–Pugh classification)   - Albumin <28 g/L (without transfusion in the preceding 14 days) - Past or current history of neoplasm other than HCC, except for curatively treated non-melanoma skin cancer, *in situ* carcinoma of the cervix, or other cancer curatively treated and with no evidence of disease for ≥5 years - Known central nervous system or brain metastasis that was either symptomatic or untreated - Medical history of difficulty swallowing, malabsorption, or other chronic GI disease, or conditions that could have hampered compliance and/or absorption of the tested products - Clinically significant GI bleeding within 4 weeks before day 1 of study treatment - Impaired cardiac function:   - Left ventricular ejection fraction <45% on recent echocardiography^‡^   - Serious arrhythmia   - Unstable angina pectoris   - Congestive heart failure New York Heart Association III and IV   - Myocardial infarction within the 12 months before day 1 of study treatment   - Pericardial effusion - Hypertension uncontrolled by standard therapies (not stabilized to ≤150/90 mmHg) - Family history of long QT syndrome, or treatment with any agent known to prolong QT/QTc interval, or with a marked prolongation of QT/QTc interval (e.g. repeated demonstration of a QTc interval >450 ms) - Known human immunodeficiency virus infection - Acute pancreatitis and/or chronic pancreatitis, with elevated lipase and/or amylase, clinical symptoms, and/or imaging studies indicative of the diagnosis (mainland Chinese patients only) - Known or suspected drug hypersensitivity to any ingredients of tepotinib - Pregnancy or lactation (female patients only), or patients of reproductive potential not willing or able to employ a highly effective method of birth control/contraception to prevent pregnancy from 2 weeks before receiving study drug until 3 months after receiving the last dose of study drug. A highly effective method of contraception was defined as having a low failure rate (<1% per year) when used consistently and correctly - Concurrent treatment with a non-permitted drug - Substance abuse, other acute or chronic medical or psychiatric condition, or laboratory abnormalities that could have increased the risk associated with study participation in the opinion of the investigator - Participation in another interventional clinical study within the 28 days before day 1 of study treatment or within a time period that was less than the cycle length for the investigational treatment (whichever was shorter), or if the patient had any adverse event caused by the investigational treatment that was not recovered to grade 1 or less - Previous anticancer treatment-related toxicities not recovered to baseline or grade 0 to 1 (except alopecia) - Any concurrent medical condition or disease that would potentially compromise the conduct of the study (at the discretion of the investigators) - Clinically significant third space fluid accumulation despite the use of diuretics (e.g. moderate-to-large ascites that required tapping, or pleural effusion that either required tapping or resulted in shortness of breath) - Complete occlusion of the major portal vein or vena cava due to HCC (the major portal vein was defined as the part of portal vein between the union of the splenic and superior mesenteric veins and the first bifurcation into the left and right vein) | |
| **Phase Ib only** | **Phase II only** |
| *No additional exclusion criteria* | - Prior systemic anticancer treatment for advanced HCC, including targeted therapy (e.g. sorafenib), chemotherapy, or any other investigational agent - Known or suspected drug hypersensitivity to any ingredients of sorafenib |

*Local-regional therapy was not to contain sorafenib; ^†^Palliative bone-directed radiotherapy was to be within a limited field of radiation and for palliation only; it was to be a short course, according to local institutional recommendations, and to be completed ≥7 days before the first administration of study treatment; ^‡^A screening left ventricular ejection fraction assessment without history of congestive heart failure was not required unless clinically indicated)

*BCLC* Barcelona Clinic liver cancer, *CrCl* creatinine clearance, *ECOG PS* Eastern Cooperative Oncology Group performance status, *GI* gastrointestinal, *IHC* immunohistochemistry, *PD* progressive disease, *RECIST* Response Evaluation Criteria in Solid Tumors, *ULN* upper limit of normal.

**Supplementary Table 3**. DLT criteria in Phase Ib

| DLT criteria |
| --- |
| Any of the following toxicities at any dose level and judged to be related to trial treatment by the investigator:   - Grade 4 neutropenia for >7 days - Grade ≥3 febrile neutropenia for >1 day - Grade 4 thrombocytopenia or grade 3 with non-traumatic bleeding - Grade ≥3 nausea/vomiting and/or diarrhea despite optimal treatment - Any grade ≥3 non-hematologic event except aforementioned gastrointestinal events and alopecia. Specific definitions existed for the following cases:   - Grade ≥3 liver AE requiring a recovery period of more than 7 days to the baseline or to grade 1 (or less; this criterion was not limited to liver function tests)   - Grade ≥3 lipase and/or amylase elevation with confirmation of pancreatitis, either based on clinical or radiological signs was not considered as a DLT. An isolated lipase and/or amylase elevation of grade ≥3 without clinical or radiological evidence of pancreatitis was not classified as a DLT |

*AE* adverse event, *DLT* dose-limiting toxicity.

**Supplementary Table 4**. Baseline characteristics (Phase Ib study)

|  | **Tepotinib** | | | **Total *n* = 27** |
| --- | --- | --- | --- | --- |
|  | **300 mg *n* = 7** | **500 mg *n* = 14** | **1000 mg *n* = 6** |  |
| Male:female, *n* | 6:1 | 12:2 | 5:1 | 23:4 |
| Median (range) age, years | 61.0  (38–69) | 56.0  (38–67) | 55.5  (43–69) | 57.0  (38–69) |
| ECOG PS 0/1, *n* | 4/3 | 5/9 | 2/4 | 11/16 |
| HBV test*, *n* (%)  Positive  Negative | 4 (57.1) 2 (28.6) | 8 (57.1) 4 (28.6) | 3 (50.0) 2 (33.3) | 15 (55.6) 8 (29.6) |
| HCV test, *n* (%)  Positive  Negative | 2 (28.6) 5 (71.4) | 2 (14.3) 12 (85.7) | 1 (16.7) 5 (83.3) | 5 (18.5) 22 (81.5) |
| Macroscopic tumor, *n* (%)  Diffuse  Nodular  Other  Not available | 1 (14.3)  2 (28.6)  2 (28.6)  2 (28.6) | 0 (0.0)  8 (57.1)  4 (28.6)  2 (14.3) | 0 (0.0)  2 (33.3)  2 (33.3)  2 (33.3) | 1 (3.7)  12 (44.4)  8 (29.6)  6 (22.2) |
| BCLC stage, *n* (%)  C | 7 (100.0) | 14 (100.0) | 6 (100.0) | 27 (100.0) |
| Prior therapy,^†^ *n* (%)  Drug  Loco-regional  Radiotherapy  Surgery | 6 (85.7)  6 (85.7)  0 (0.0)  3 (42.9) | 14 (100.0)  11 (78.6)  7 (50.0)  11 (78.6) | 6 (100.0)  4 (66.7)  5 (83.3)  4 (66.7) | 26 (96.3)  21 (77.8)  12 (44.4)  18 (66.7) |
| Lines of prior therapy, *n* (%)  1  2  ≥3 | 3 (42.9)  3 (42.9)  0 (0.0) | 6 (42.9)  4 (28.6)  4 (28.6) | 4 (66.7)  0 (0.0)  2 (33.3) | 13 (48.1)  7 (25.9)  6 (22.2) |
| Prior sorafenib, *n* (%) | 4 (57.1) | 12 (85.7) | 3 (50.0) | 19 (70.4) |
| Baseline AFP ≥200 IU/mL, *n* (%) | 4 (57.1) | 9 (64.3) | 2 (33.3) | 15 (55.6) |

*HBV testing was not mandatory in Phase Ib.

*AFP* alpha-fetoprotein, *BCLC* Barcelona Clinic liver cancer, *ECOG* Eastern Cooperative Oncology Group, *HBV* hepatitis B virus.

**Supplementary Table 5**. List of institutional review boards or independent ethics committees of each center

| **Site No.** | **Investigator Name** | **IRB/IEC Name and Address** | **Chairperson (if known)** | **IRB reference number** |
| --- | --- | --- | --- | --- |
| 101 | Dr Jianming Xu | Ethical Committee of Affiliated  Hospital of Academy of Military Medical Sciences  No.8 Dongda Street  Fengtai District  Beijing  China - 100071 | Not Available |  |
| 102 | Dr Xi Chen | EC of Fuzhou General Hospital of  Nanjing Military Area Command of Chinese PLA  No.156 Xierhuan North Road  Fuzhou  China, 350025 | Not Available |  |
| 103 | Dr Zhiqiang Meng | Fudan University Shanghai Cancer Center  No. 270 Dongan Road  Xuhui District  Shanghai  China, 200032 | Not Available |  |
| 105 | Dr Ying Cheng | EC of Jilin Cancer Hospital  No. 1018 Huguang Road  Chaoyang District  Changchun, Jilin  China 130012 | Not Available |  |
| 106 | Dr Hongming Pan | EC of Hangzhou Sir Runrun Shaw  Hospital  No. 3 Chundong Road  Hangzhou  Zhejiang  China, 310016 | Not Available |  |
| 107 | Dr Shukui Qin | EC of The 81st Hospital of PLA  NO.34, 34 Biao Yanggongjing  Nanjing  Jiangsu China | Not Available |  |
| 108 | Dr Yabing Guo | EC of Nanfang Hospital of  Southern Medical University  16th Floor, New Laboratory Building  No. 1838, North Guangzhou Avenue, Guangzhou  Guangdong  China 510515 | Xun Zhang |  |
| 110 | Dr Minshan Chen | EC of Sun Yat-sen University, Cancer Center  8^th^ Floor, No.1 Building  651 Dongfeng Road East  Yuexiu District  Guangzhou  China 510060 | Wangqing Peng |  |
| 111 | Dr Zhenggang Ren | EC of Zhongshan Hospital  Fudan University  NO.180 Fenglin Road  Xuhui District  Shanghai  China | Not Available |  |
| 112 | Dr Bangwei Cao | EC of Beijing Friendship Hospital  Capital Medical University  No.95 Yong'an Road, Xicheng District  Beijing  China - 100050 | Not Available |  |
| 113 | Dr Yuxian Bai | EC of Harbin Medical University  Cancer Hospital  No. 150 Haping Road  Nangang District  Harbin  Heilongjiang Province  China -150081 | Not Available |  |
| 114 | Dr Yongqian Shu | EC of Nanjing First Hospital  Affiliated to Nanjing Medical University  No. 300 Guangzhou Road  Nanjing, Jiangsu  China, 210029 | Not Available |  |
| 115 | Dr Tianqiang Song | EC of Tianjin Cancer Hospital  Tiyuan North, Huanhuxi Road  Tianjin  China, 300060 | Not Available |  |
| 117 | Dr Peiguo Cao | EC of The Third Xiangya Hospital of Central South University  No. 138 Tongzipo Road  Hexi Yuelu District  Changsha, Hunan  China, 410013 | Not Available |  |
| 118 | Dr Chunyi Hao | EC of Beijing Cancer Hospital  No. 52 Fucheng Road  Haidian District  Beijing  China 100142 | Not Available |  |
| 201 | Dr Yee Chao | Taipei Veterans General Hospital  Institutional Review Board  No.201, Sec. 2, Shipai Road  Beitou District, Taipei City 112  Taiwan, 11217, ROC | Shinn-Jang Hwang, MD |  |
| 202 | Dr Teng-Yu Lee | Taichung Veterans General Hospital  Institutional Review Board  No.1650 Taiwan Boulevard Sect. 4  Taichung  Taiwan 40705, ROC | Jiaan-Der Wang, MD, PhD |  |
| 203 | Dr Ann-Lii Cheng | National Taiwan University Hospital  Research Ethics Committee  No.7, Chung-Shan South Road  Taipei  Taiwan 100, ROC 10002 | Fu-Chang Tsai, MD |  |
| 204 | Dr Chia-Jui Yen | National Cheng Kung University Hospital  Institutional Review Board  138 Sheng-Li Road  Tainan 704  Taiwan | Thy-Sheng Lin MD |  |
| 205 | Dr Tsang-En Wang | Mackay Memorial Hospital  Institutional Review Board  No.92, Sec.2, Zhong-shan N Road  Taipei  Taiwan 10449 | Yi-Shing Leu, MD |  |
| 206 | Dr Shi-Ming Lin | Chang Gung Medical Foundation  Institutional Review Board  199 Tung-Hwa North Road  Taipei  Taiwan, 10507 | Tsang-Tang Hsieh |  |
| 207 | Dr Chao-Jung Tsao | Chi Mei Medical Center  Institutional Review Board  4F, Third Medical Building  No.901, Zhonghua Road  Yongkang District 710  Taiwan | Chung-Hsi Hsing, MD |  |
| 208 | Dr Kun-Ming Rau | Chang Gung Medical Foundation  Institutional Review Board  199 Tung-Hwa North Road  Taipei  Taiwan, 10507 | Tsang-Tang Hsieh |  |
| 209 | Dr Hsu-Heng Yen | Changhua Christian Hospital Institutional Review Board  135 Nanxiao Street Changhua City  Changhua County 500  Taiwan | KunTu Yeh, MD |  |
| 210 | Dr Shinn-Cherng Chen | Kaohsiung Medical University Chung-Ho Memorial Hospital  Institutional Review Board  No. 100, Tzyou 1^st^ Road  Kaohsiung 807  Taiwan | Dr. Li-Tzong Chen,  MD, PhD |  |
| 301 | Dr Baek-Yeol Ryoo | IRB of Asan Medical Center  Asan Medical Center Institutional Review Board  88, Olympic-ro 43-gil  Songpa-gu,  Seoul, 05505  Korea | Not Available |  |
| 302 | Dr Hye Jin Choi | Institutional Review Board of  Severance Hospital or Human  Research Protection Center of Severance Hospital  50 Yonsei-Ro  Seodaemun-gu  Seoul, 03722  Korea | Seung Min Kim |  |
| 303 | Dr Tae-You Kim | IRB of Seoul National University Hospital  101, Daehak-ro  Jongno-gu  Seoul, 03080  Korea | Not Available |  |
| 304 | Dr Ho Yeong Lim | Samsung Medical Center Officer of  Human Research Protection  81, Irwon-ro  Gangnam-Gu  Seoul, 06351  Korea | Suk-Koo Lee |  |
| 305 | Dr Jong Young Choi | IRB of The Catholic University of Korea  Seoul St. Mary's Hospital  222 Banpo-daero, Seocho-gu  Seoul, 06591  Korea | Not Available |  |
| 306 | Dr Yeul Hong Kim | IRB of Korea University Anam Hospital  73, Inchon-ro, Seongbuk-Gu  Seoul, 02841  Korea | Not Available |  |
| 307 | Dr Joong-Won Park | IRB of National Cancer Center  323, Ilsan-ro, Ilsandong-gu  Goyang-si,  Gyeonggi-do, 10408  Korea | Not Available |  |
| 308 | Dr Jin Young Kim | IRB of Keimyung University Dongsan Hospital  56, Dalseong-ro, Jung-gu  Daegu, 41931  Korea | Not Available |  |
| 309 | Dr Won Young Tak | IRB of Kyungpook National University Hospital  130 Dongduk-ro, Jung-gu  Daegu, 41944  Korea | Not Available |  |
| 310 | Dr Jeong Heo | IRB of Pusan National University Hospital  179, Gudeok-Ro, Seo-Gu  Busan, 49241  Korea | Not Available |  |
| 311 | Dr Jee Hyun Kim | IRB of Seoul National University  Bundang Hospital  82, Gumi-ro 173 Beon-gil  Bundang-gu, Seongnam-si  Gyeonggi-do, 13620  Korea | Not Available |  |
| 312 | Dr Byung-Ho Kim | IRB of Kyung Hee University Hospital  23, Kyungheedae-ro  Dongdaemun-gu  Seoul 02447  Korea | Not Available |  |
| 313 | Dr Mong Cho | IRB of Pusan National University Yangsan Hospital  20, Geumo-ro, Mulgeum-eup  Yangsan-si  Gyeongsangnam-do,50612  Korea | Not Available |  |
| 314 | Dr Jae Youn Cheong | IRB of Ajou University Hospital  164, World cup-ro  Yeongtong-gu, Suwon-si  Gyeonggi-do, 16499  Korea | Not Available |  |
| 315 | Dr Kwan Sik Lee | Institutional Review Board of  Gangnam Severance Hospital  Yonsei University Health System or Human Research Protection  Center of Gangnam Severance Hospital  Yonsei University Health System  211 Eonju-ro  Gangnam-gu  Seoul, 06273  Korea | Joon-Seong Park |  |
| 316 | Dr Hongjae Chon | Institutional Review Board of CHA  Bundang Medical Center  CHA University  59, Yatap-ro, Bundang-gu  Seongnam-si  Gyeonggi-do,13496  Korea | Sung Won Kwon |  |
| 317 | Dr Ji Hoon Kim | IRB of Korea University Guro Hospital  148, Gurodong-ro, Guro-gu  Seoul, 08308  Korea | Not Available |  |
| 318 | Dr Sang Young Han | IRB of Dong-A University Medical Center  26, Daeshingongwon-ro  Seo-gu  Busan, 49201,  Korea | Not Available |  |

**Supplementary Figure 1**. Phase II study design


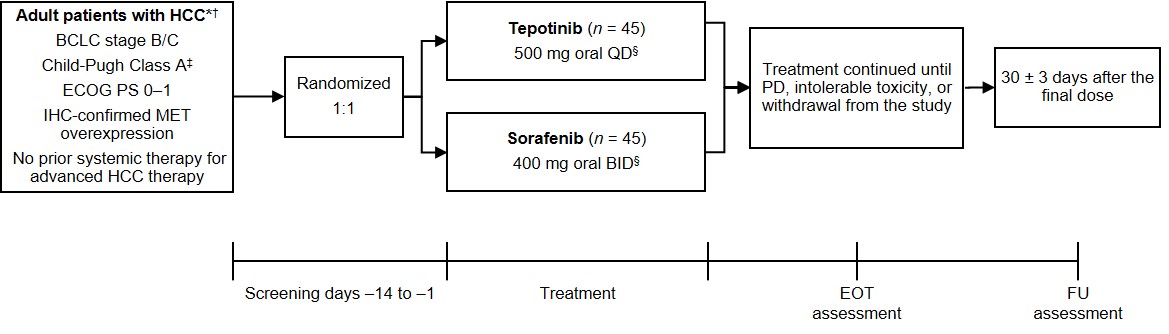


*From 43 sites in China, South Korea, and Taiwan; ^†^Histologically or cytologically confirmed; ^‡^Without encephalopathy; ^§^In continuous 21-day cycles.

MET overexpression (Phase II only), as determined by the central laboratory, was defined as moderate (2+) or strong (3+) staining intensity for MET using IHC in the majority (≥50%) of tumor cells.

*BCLC* Barcelona Clinic liver cancer, *BID* twice daily, *ECOG PS* Eastern Cooperative Oncology Group performance status, *EOT* end of trial, *FU* follow-up, *IHC* immunohistochemistry, *PD* progressive disease, *QD* once daily.

**Supplementary Figure 2.** Patient disposition during **a** Phase Ib and **b** Phase II parts of the study

**a**


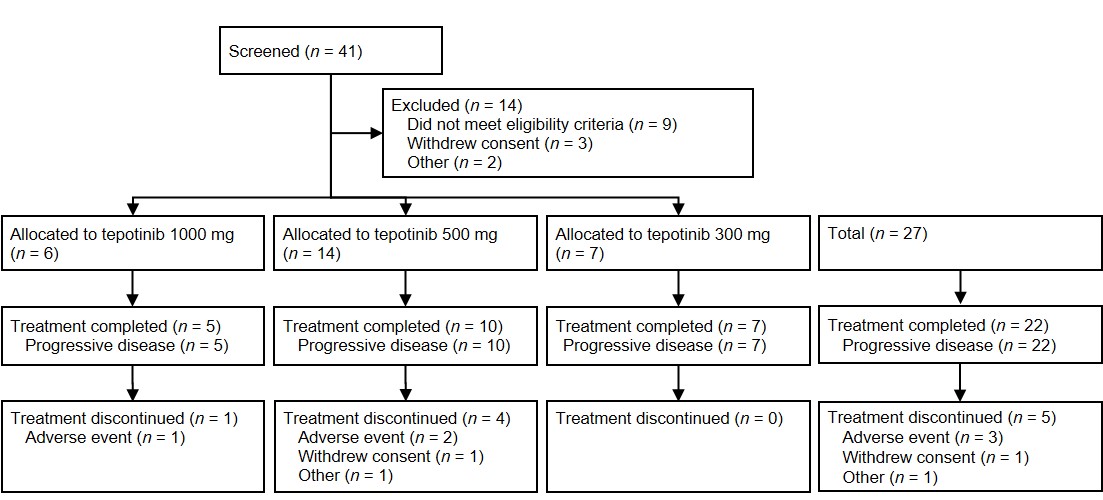


**b**

**
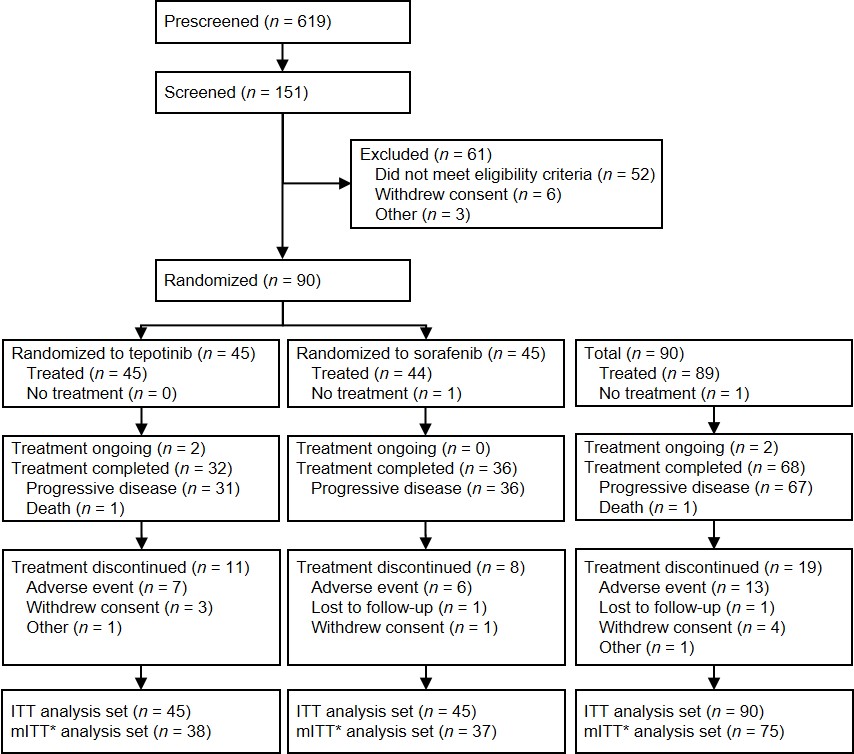
**

*All patients with HCC with MET overexpression who were randomized to study treatment.

*ITT* intention-to-treat, *mITT* modified intention-to-treat.
